# Supplementary material for: The ABCG2 Q141K hyperuricemia and gout associated variant illuminates the physiology of human urate excretion
Source: Nat Commun. 2020 Jun 2;11:2767. doi: 10.1038/s41467-020-16525-w (PMC7265540; doi:10.1038/s41467-020-16525-w)
Supplement: Supplementary file 1 — Supplementary Information [file 41467_2020_16525_MOESM1_ESM.pdf]

The *ABCG2* Q141K hyperuricemia and gout associated variant illuminates the physiology of human urate excretion

Hoque *et al.*

Supplementary Information

Supplementary Figure 1: Interventional study extended data

A: Analysis stratified by ancestry

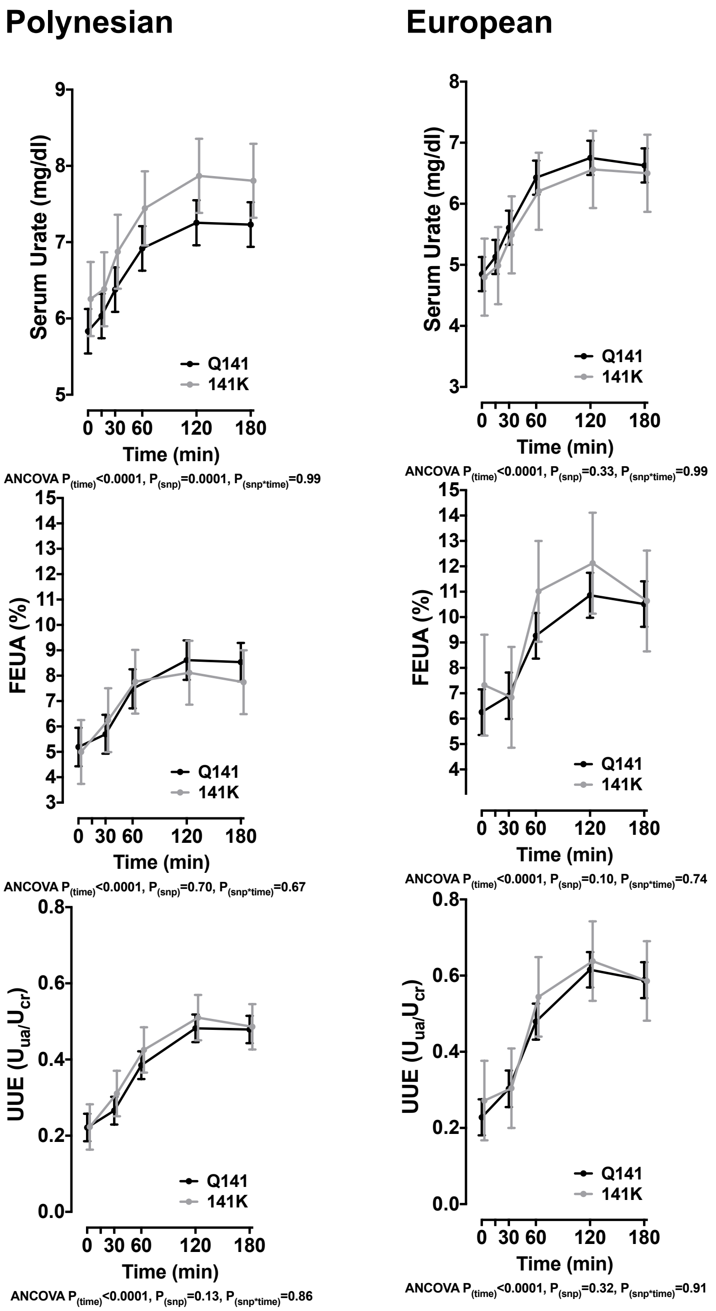

Supplementary Figure 1:

(A) Effect of *ABCG2* genotypes (Q141, n=79 and 141K, n=21 participants, Supplementary Table 1 for characteristics of participants), stratified by ancestry, on dependence of serum urate (SU), FEUA, and urinary urate excretion (UUE) in participants of either Polynesian or European ancestry following inosine load (+/- standard deviation of the mean [SD]). Statistical significance evaluated by a two-tailed ANCOVA, adjusted for age, sex, and BMI. Source data are provided as a Source Data file.

Supplementary Figure 1: Interventional study extended data (continued)

B: Relationship between SU and FEUA in Q141 and 141K individuals

Total

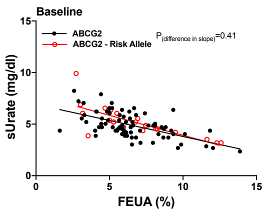

	FEUA (%) vs. ABCG2	FEUA (%) vs. ABCG2 - Risk Allele
Pearson r		
r	-0.587	-0.7493
95% confidence interval	-0.716 to -0.4192	-0.8924 to -0.4695
R squared	0.3446	0.5615
P value		
P (two-tailed)	<0.0001	<0.0001
P value summary	****	****
Significant? (alpha = 0.05)	Yes	Yes
Number of XY Pairs	78	21

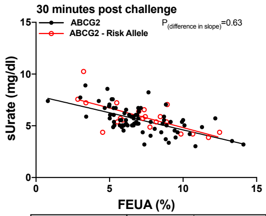

	FEUA (%) vs. ABCG2	FEUA (%) vs. ABCG2 - Risk Allele
Pearson r		
r	-0.6188	-0.6812
95% confidence interval	-0.7409 to -0.4572	-0.86 to -0.3534
R squared	0.383	0.464
P value		
P (two-tailed)	<0.0001	0.0007
P value summary	****	***
Significant? (alpha = 0.05)	Yes	Yes
Number of XY Pairs	76	21

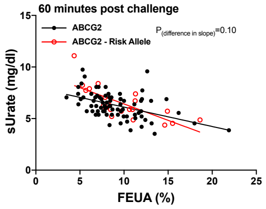

	FEUA (%) vs. ABCG2	FEUA (%) vs. ABCG2 - Risk Allele
Pearson r		
r	-0.4722	-0.7387
95% confidence interval	-0.6296 to -0.2775	-0.8874 to -0.4507
R squared	0.2229	0.5457
P value		
P (two-tailed)	<0.0001	0.0001
P value summary	****	***
Significant? (alpha = 0.05)	Yes	Yes
Number of XY Pairs	77	21

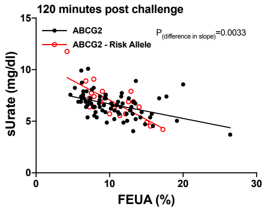

	FEUA (%) vs. ABCG2	FEUA (%) vs. ABCG2 - Risk Allele
Pearson r		
r	-0.4515	-0.7571
95% confidence interval	-0.6135 to -0.2531	-0.896 to -0.4834
R squared	0.2039	0.5732
P value		
P (two-tailed)	<0.0001	<0.0001
P value summary	****	****
Significant? (alpha = 0.05)	Yes	Yes
Number of XY Pairs	77	21

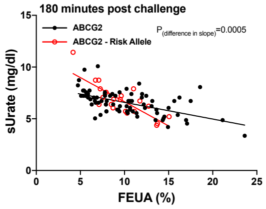

	FEUA (%) vs. ABCG2	FEUA (%) vs. ABCG2 - Risk Allele
Pearson r		
r	-0.5577	-0.7971
95% confidence interval	-0.6941 to -0.3826	-0.9142 to -0.5571
R squared	0.311	0.6354
P value		
P (two-tailed)	<0.0001	<0.0001
P value summary	****	****
Significant? (alpha = 0.05)	Yes	Yes
Number of XY Pairs	78	21

Polynesian

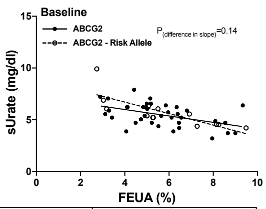

	FEUA (%) vs. ABCG2	FEUA (%) vs. ABCG2 - Risk Allele
Pearson r		
r	-0.4747	-0.7857
95% confidence interval	-0.6923 to -0.1781	-0.9329 to -0.4139
R squared	0.2254	0.6174
P value		
P (two-tailed)	0.0030	0.0015
P value summary	**	**
Significant? (alpha = 0.05)	Yes	Yes
Number of XY Pairs	37	13

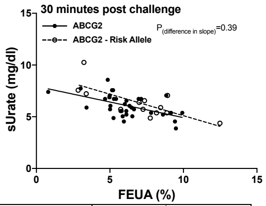

	FEUA (%) vs. ABCG2	FEUA (%) vs. ABCG2 - Risk Allele
Pearson r		
r	-0.5468	-0.7485
95% confidence interval	-0.742 to -0.2661	-0.92 to -0.3361
R squared	0.299	0.5602
P value		
P (two-tailed)	0.0006	0.0032
P value summary	***	**
Significant? (alpha = 0.05)	Yes	Yes
Number of XY Pairs	36	13

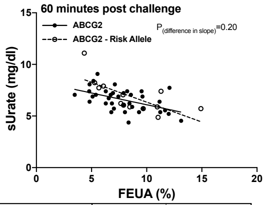

	FEUA (%) vs. ABCG2	FEUA (%) vs. ABCG2 - Risk Allele
Pearson r		
r	-0.5036	-0.7057
95% confidence interval	-0.714 to -0.2098	-0.9049 to -0.2532
R squared	0.2536	0.498
P value		
P (two-tailed)	0.0017	0.0070
P value summary	**	**
Significant? (alpha = 0.05)	Yes	Yes
Number of XY Pairs	36	13

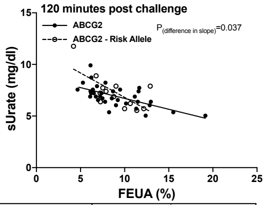

	FEUA (%) vs. ABCG2	FEUA (%) vs. ABCG2 - Risk Allele
Pearson r		
r	-0.6372	-0.6914
95% confidence interval	-0.8005 to -0.3858	-0.8997 to -0.2268
R squared	0.406	0.478
P value		
P (two-tailed)	<0.0001	0.0089
P value summary	****	**
Significant? (alpha = 0.05)	Yes	Yes
Number of XY Pairs	35	13

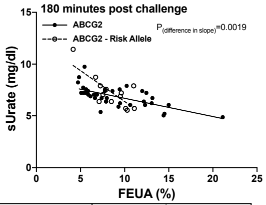

	FEUA (%) vs. ABCG2	FEUA (%) vs. ABCG2 - Risk Allele
Pearson r		
r	-0.6572	-0.7566
95% confidence interval	-0.809 to -0.4234	-0.9229 to -0.3526
R squared	0.432	0.5725
P value		
P (two-tailed)	<0.0001	0.0028
P value summary	****	**
Significant? (alpha = 0.05)	Yes	Yes
Number of XY Pairs	37	13

European

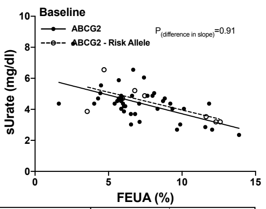

	FEUA (%) vs. ABCG2	FEUA (%) vs. ABCG2 - Risk Allele
Pearson r		
r	-0.5635	-0.701
95% confidence interval	-0.7424 to -0.3094	-0.9409 to 0.007195
R squared	0.3175	0.4914
P value		
P (two-tailed)	0.0001	0.0527
P value summary	***	ns
Significant? (alpha = 0.05)	Yes	No
Number of XY Pairs	41	8

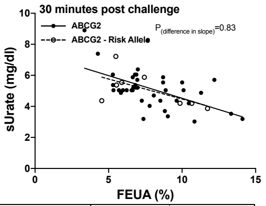

	FEUA (%) vs. ABCG2	FEUA (%) vs. ABCG2 - Risk Allele
Pearson r		
r	-0.5544	-0.6216
95% confidence interval	-0.7384 to -0.2936	-0.9223 to 0.1477
R squared	0.3074	0.3864
P value		
P (two-tailed)	0.0002	0.0999
P value summary	***	ns
Significant? (alpha = 0.05)	Yes	No
Number of XY Pairs	40	8

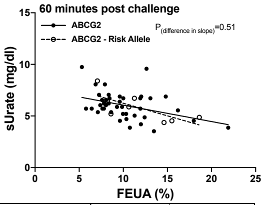

	FEUA (%) vs. ABCG2	FEUA (%) vs. ABCG2 - Risk Allele
Pearson r		
r	-0.3961	-0.7322
95% confidence interval	-0.6273 to -0.1007	-0.9478 to -0.05689
R squared	0.1569	0.5361
P value		
P (two-tailed)	0.0104	0.0389
P value summary	*	*
Significant? (alpha = 0.05)	Yes	Yes
Number of XY Pairs	41	8

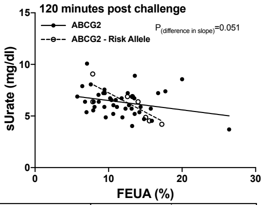

	FEUA (%) vs. ABCG2	FEUA (%) vs. ABCG2 - Risk Allele
Pearson r		
r	-0.2828	-0.8117
95% confidence interval	-0.5403 to 0.02312	-0.9646 to -0.25
R squared	0.07997	0.6588
P value		
P (two-tailed)	0.0696	0.0144
P value summary	ns	*
Significant? (alpha = 0.05)	No	Yes
Number of XY Pairs	42	8

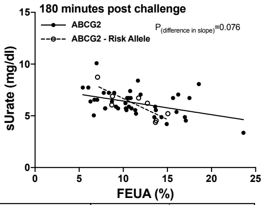

	FEUA (%) vs. ABCG2	FEUA (%) vs. ABCG2 - Risk Allele
Pearson r		
r	-0.4283	-0.8231
95% confidence interval	-0.6502 to -0.1389	-0.9669 to -0.2819
R squared	0.1834	0.6774
P value		
P (two-tailed)	0.0052	0.0121
P value summary	**	*
Significant? (alpha = 0.05)	Yes	Yes
Number of XY Pairs	41	8

Supplementary Figure 1 (continued):

(B) Effect of *ABCG2* genotypes on dependence of SU on FEUA stratified by ancestry, Polynesian or European, adjusted for age, sex, and BMI. Each SU and commensurate FEUA measured at each of 5 time points plotted for each individual with the Q141 allele (black or solid line) or 141K (risk) allele (red or dashed line) with linear regression fits extended, comparable to the nonstratified data in Figure 1 D and E. Time points for each ancestry or total has a unique panel where the number of participants (n, or xy pair), statistical test and p value are listed. Comparison of slopes of linear regression done with two-tailed ANCOVA. Source data are provided as a Source Data file.

Supplementary Figure 1: Interventional study extended data (continued)

C: Effect of *ABCG2* genotypes on dependence of SU on FEUA, stratified by ancestry

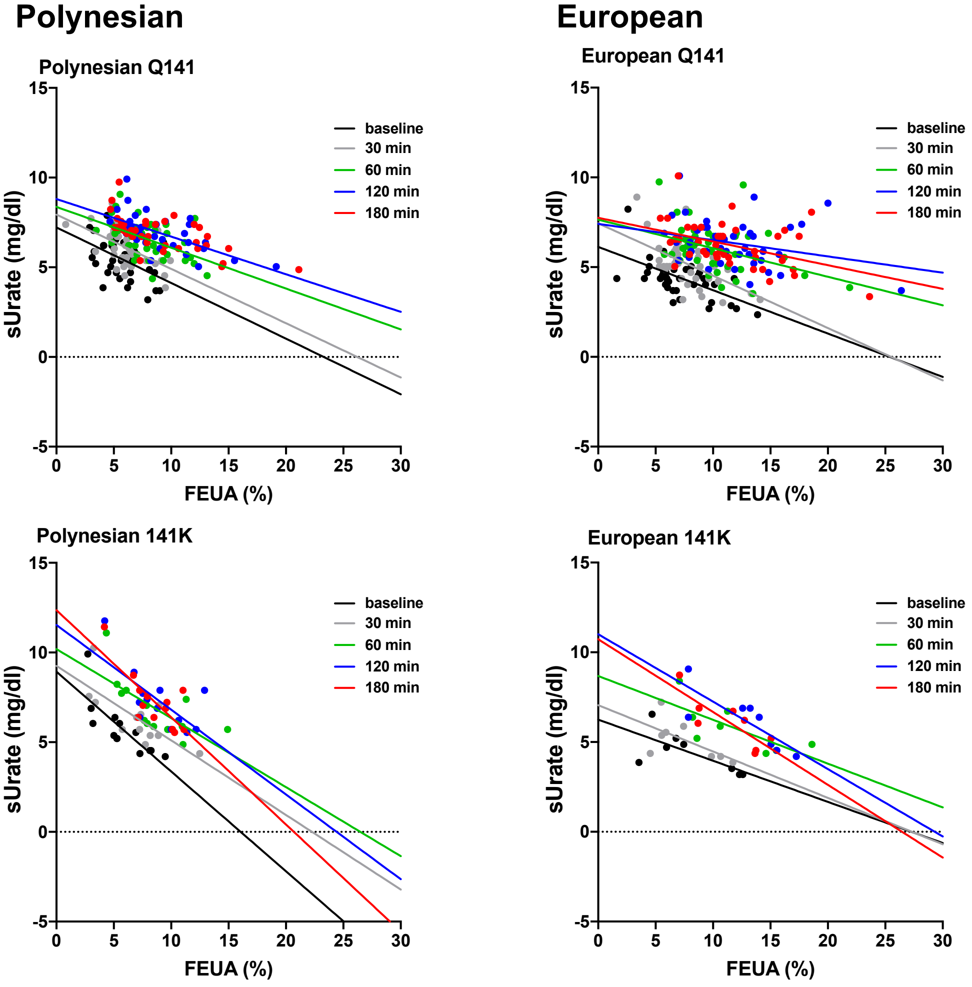

D: Effect of *ABCG2* genotypes on dependence of SU on UUE, stratified by ancestry

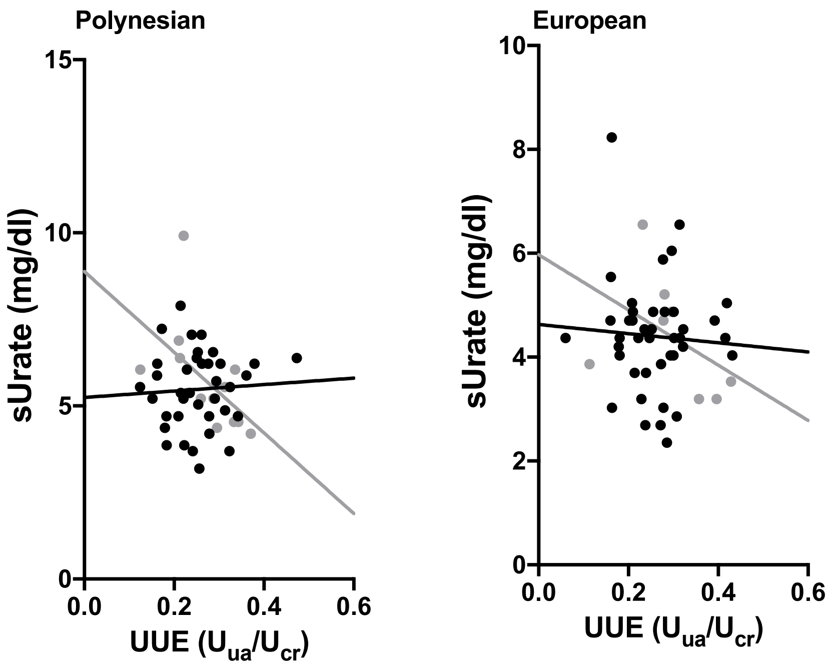

Supplementary Figure 1 (continued):

(C) Effect of *ABCG2* genotypes (Q141, n=79 and 141K, n=21 participants, Supplementary Table 1 for characteristics of participants), stratified by ancestry, on serum urate's (SU) dependence on FEUA; each SU and commensurate FEUA measured at each of 5 time points plotted for each individual with the Q141 allele or 141K (risk) allele with linear regression fits extended stratified by ancestry, Polynesian or European. (D) Effect of *ABCG2* genotypes (Q141, n=79 and 141K, n=21 participants, Supplementary Table 1 for characteristics of participants), stratified by ancestry, on serum urate (SU) dependence on UUE at baseline (time 0) plotted with linear regression fits (Q141 allele,141K allele) extended until UUE = 0. Source data are provided as a Source Data file.

Supplementary Figure 2: Interventional study extended data

A: SU and FEUA in participants with or without protective (urate-lowering) C allele of *SLC2A9* rs11942223

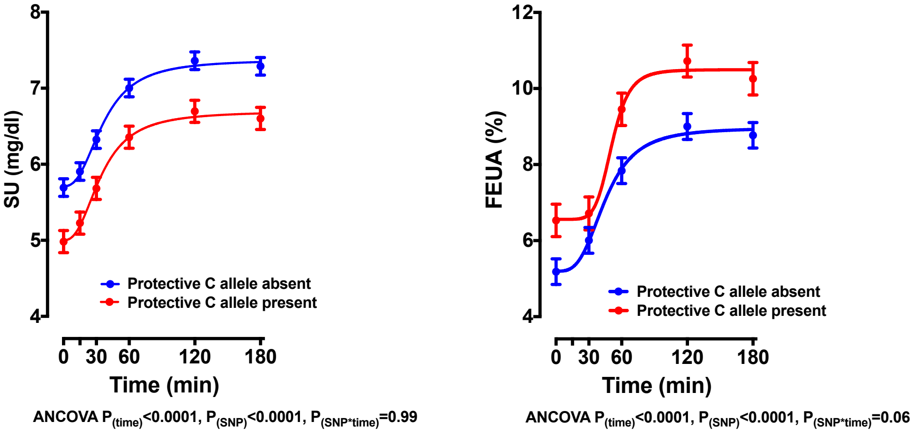

B: SU and FEUA in participants with *SLC2A9* variant stratified by ancestry

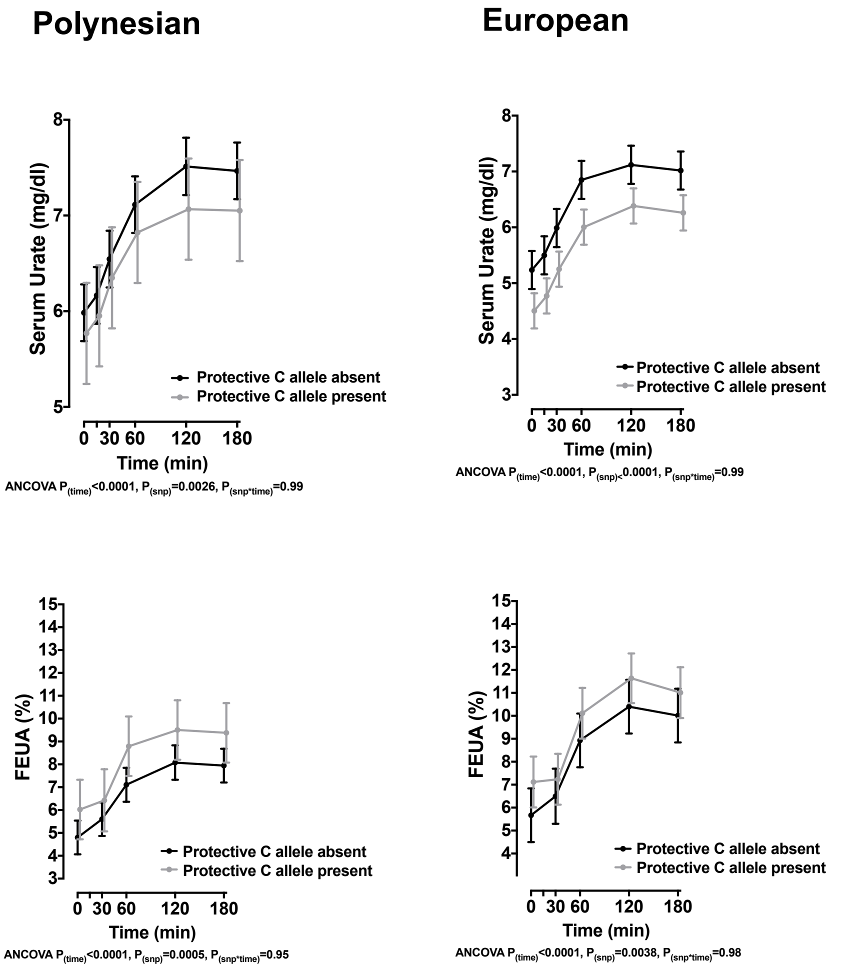

Supplementary Figure 2: Interventional study extended data

(A) Effect of *SLC2A9* genotypes (participants with [n=38] or with out [n=62] the protective, urate-lowering, C allele) on serum urate (ANCOVA: p(time)<0.0001; p(SNP)=0.0001; p(SNP*time)=0.99) and fractional excretion of urate (FEUA)(ANCOVA: p(time)<0.0001; p(SNP)=0.0001; p(SNP*time)=0.06) following inosine load to entire group (100 participants; +/- SEM). (B) Secondary analysis of *SLC2A9* genotypes, stratified by ancestry, on serum urate and FEUA in participants of either Polynesian or European ancestry following inosine load (100 participants; +/- SD of the mean). Statistical significance evaluated by a two-tailed ANCOVA, adjusted for age, sex, BMI and ancestry (A only). Source data are provided as a Source Data file.

Supplementary Figure 3: Human and mouse renal tissue data

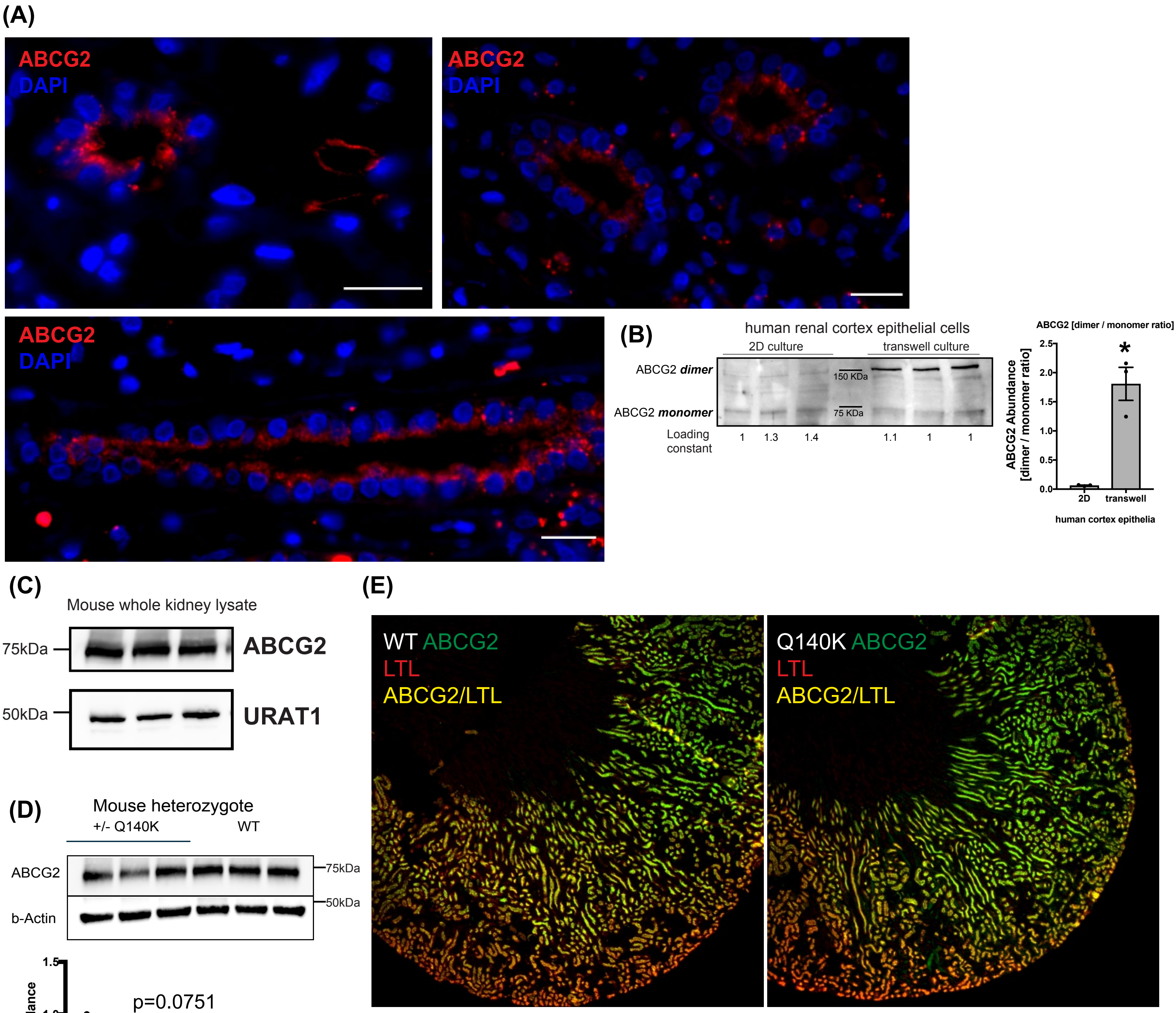

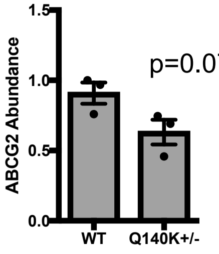

Supplementary Figure 3: Human and mouse renal tissue data
(A) Representative immunofluorescence micrographs of fixed medullary sections of human kidney samples stained for ABCG2 (red), and DAPI (blue) at 400x (scale bar 25µM)(representative of three independently repeated experiments from n=3 kidneys. (B) Western blot of cell lysate from human renal cortical epithelial cells cultured either on a flat culture dish (2D) or on a transwell, loading constant determined by total lane protein, with quantification of the dimer to monomer ratio (n=3 cultures / dishes of each, significant p=0.01, two-tailed t-test, +/-SEM). (C) Western blots of total kidney homogenate from WT (n=3) mice blotted with either ABCG2 or URAT1. (D) Relative abundance of ABCG2 protein in WT, or Q140K+/- (heterozygotic animals), n=3, p=0.0751, two-tailed T-test +/-SEM. (E) Representative (similar staining was repeated on tissue from n=3 mice) micrographs (40x; scale bar 500µM) from fixed whole mouse kidney slices stained with ABCG2 (I) in green, LTL (red), and colocalization (yellow). Source data are provided as a Source Data file.

Supplementary Figure 4: Female metabolic comparisons and ABCG2 abundance

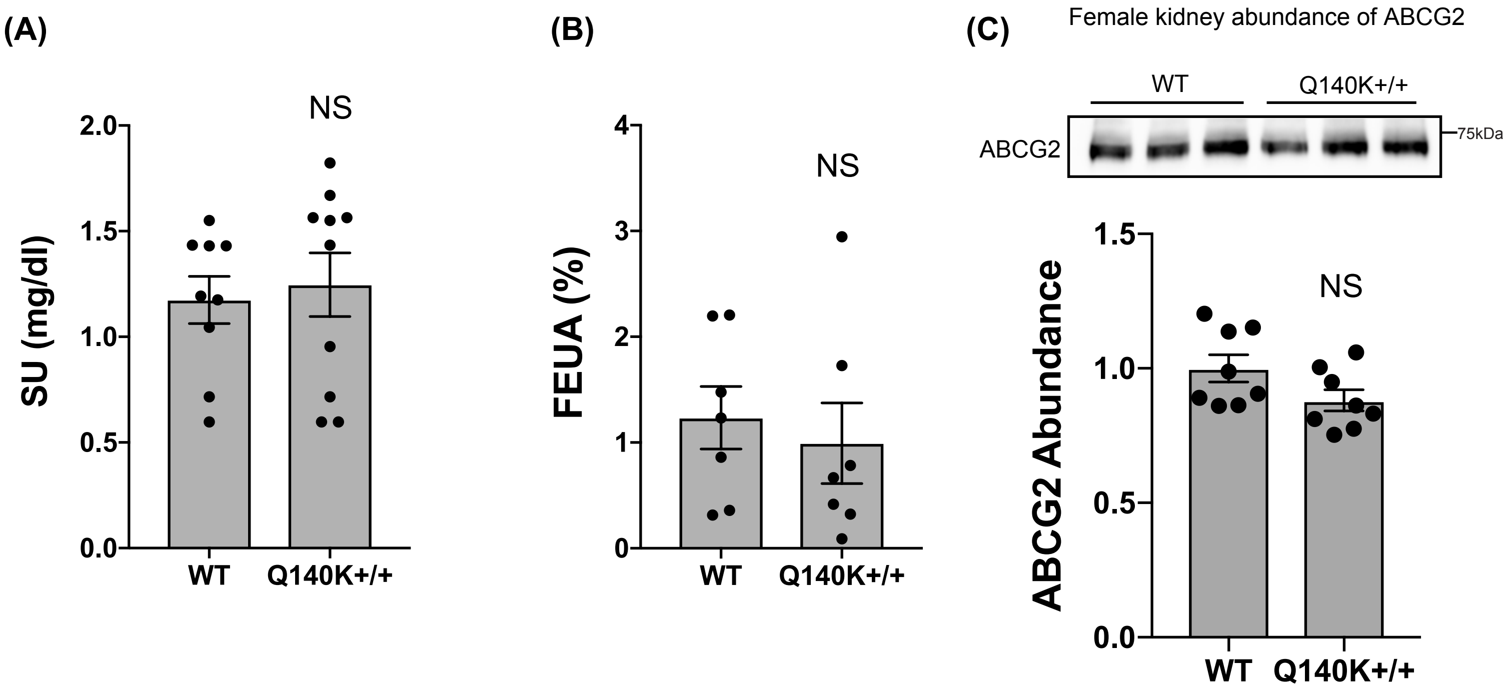

Supplementary Figure 4: Female metabolic comparisons and ABCG2 abundance
(A) Serum urate levels for female, WT, and Q140K+/+ animals (WT females n=9; Q140K+/+ n=10; p=0.7113, two-tailed Student's t-test +/-SEM). (B) Fractional excretion of urate (FEUA) measurements from female WT (n=7) and female Q140K+/+ (n=7)(p=0.6263, two-tailed Student's t-test +/-SEM). (C) Western blots of total kidney homogenate from WT (n=8) and Q140K+/+ (n=8) mice showed no change in abundance of the Q140K+/+ protein (p=0.0835, two-tailed Student's t-test; loading constant determined by total lane protein analysis [methods]+/-SEM). Source data are provided as a Source Data file.

Supplementary Figure 5: Male Q140K+/+ mice display variable fatty liver phenotype

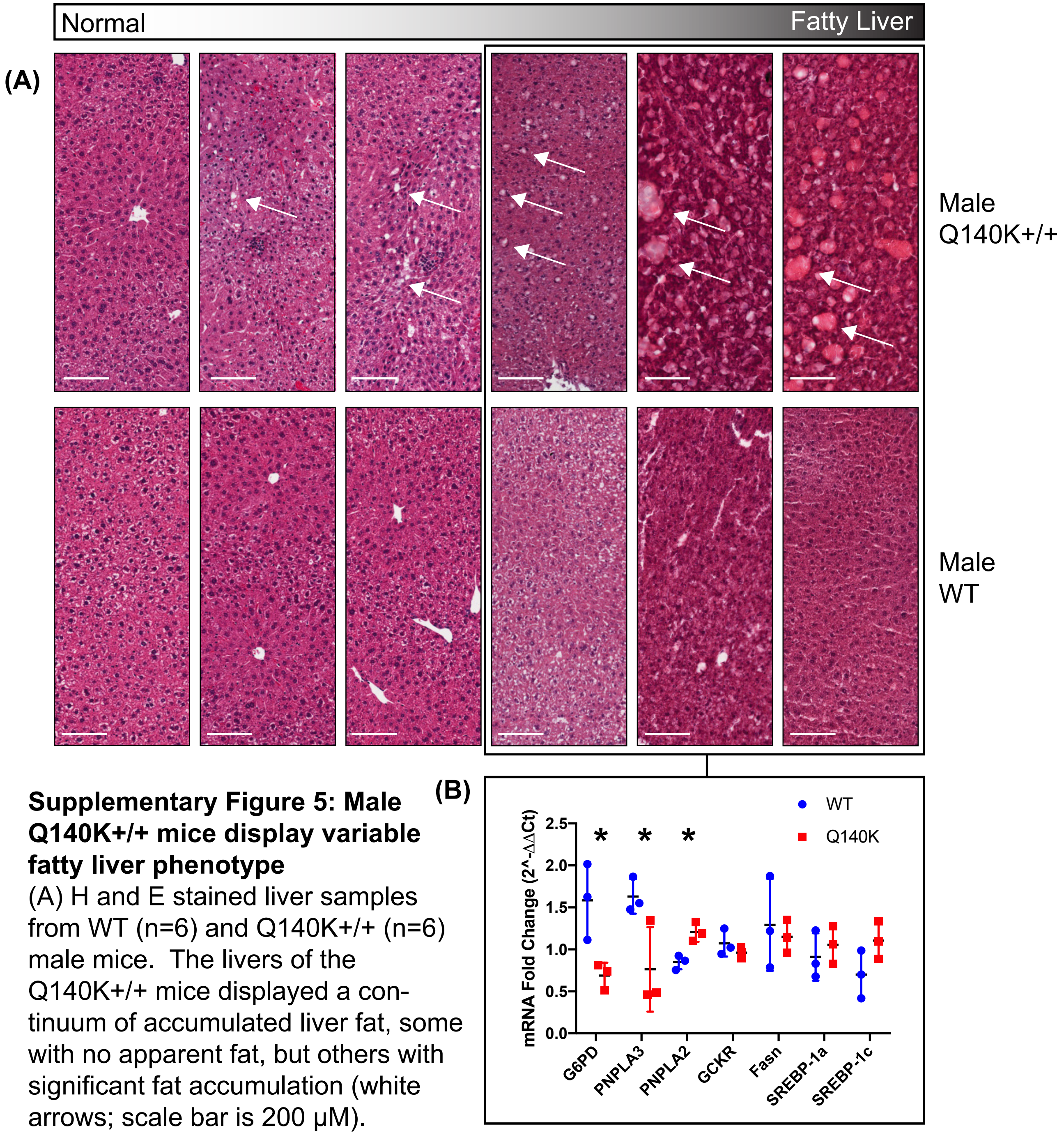

Supplementary Figure 5: Male Q140K+/+ mice display variable fatty liver phenotype

(A) H and E stained liver samples from WT (n=6) and Q140K+/+ (n=6) male mice. The livers of the Q140K+/+ mice displayed a continuum of accumulated liver fat, some with no apparent fat, but others with significant fat accumulation (white arrows; scale bar is 200 μM).

(B) Quantitative RT-PCR of 7 common genes associated with non-alchoholic fatty liver disease (NAFLD) from the three Q140K+/+ livers with significant fat accumulation (pictured in A) as compared to the matched controls revealed three genes, *G6pd (p=0.032), Pnpla3 (p=0.050), and Pnpla2 (p=0.012)* to be significantly altered (n=3 WT and Q140K+/+; two-tailed Student's t-test, +/- SEM). Source data are provided as a Source Data file.

Supplementary Figure 6: Further intestinal data from mouse model

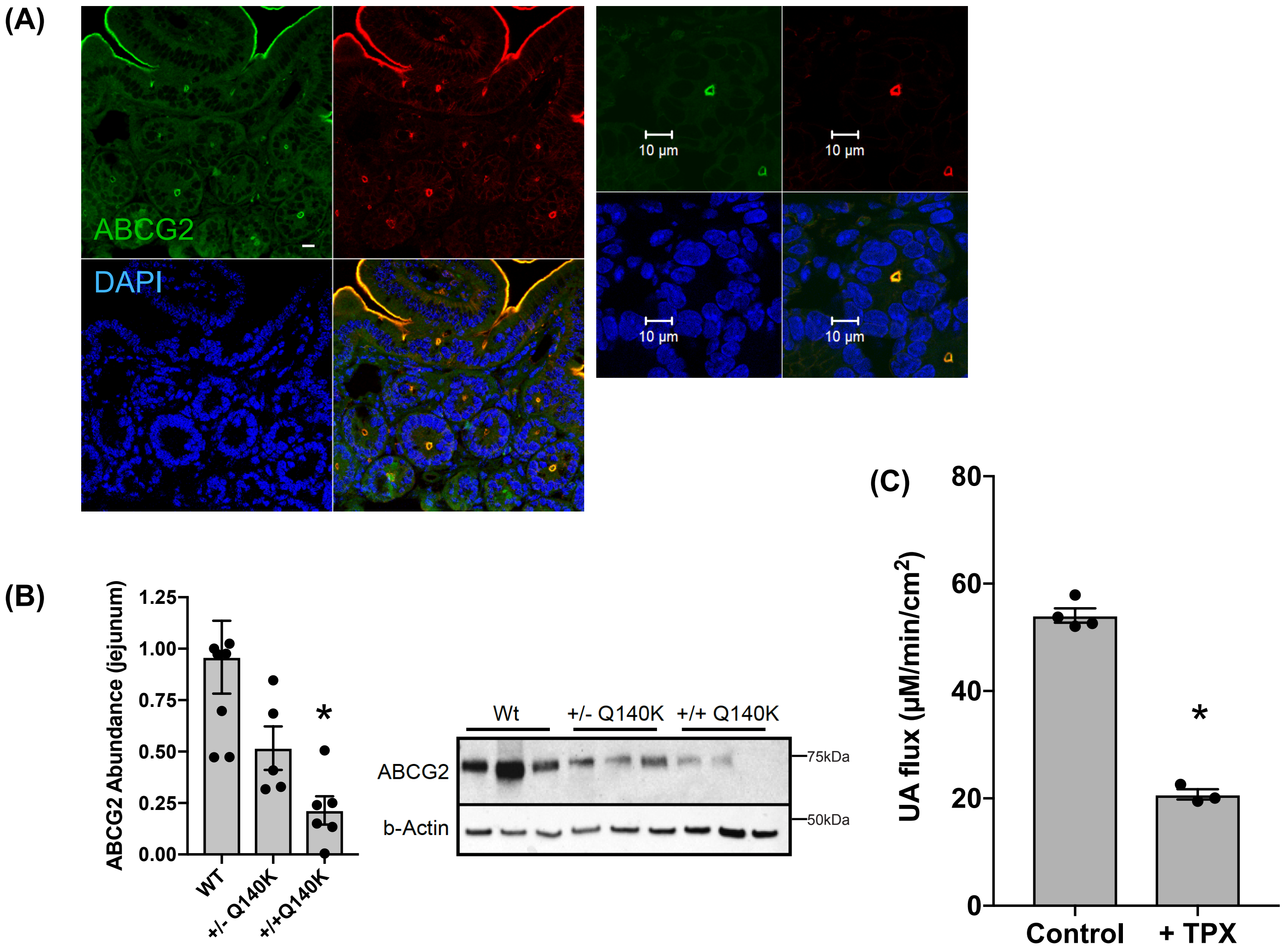

Supplementary Figure 6: Further intestinal data from mouse model

(A) Representative immunofluorescence micrographs of fixed sections of the crypt region of mouse small intestines (from n=4 mice) stained for ABCG2 (green), villi brush border marker beta-actin (red), DAPI (blue), and colocalization of ABCG2 and beta-actin (yellow) at 400x and 630x, scale bar 10μM in all images. (B) Abundance of ABCG2 in the jejunum of WT (n=8), Q140K+/- (n=5), and Q140K+/+ (n=6) male animals, and representative western blot of quantified ABCG2 abundance, loading control is beta-actin; one way ANOVA with post hoc Dunnett's test; p=0.003, +/-SEM. (C) Mean luminal flux in WT (n=3) and Q140K+/+ (n=3) jejunum loops showed different sensitivity to ABCG2 inhibitor Topiroxostat (TPX) (p<0.0001, two-tailed Student's t-test; +/-SEM). Source data are provided as a Source Data file.

Supplementary Figure 7: Partial sex stratified analysis

A: Analysis stratified by sex- Female only

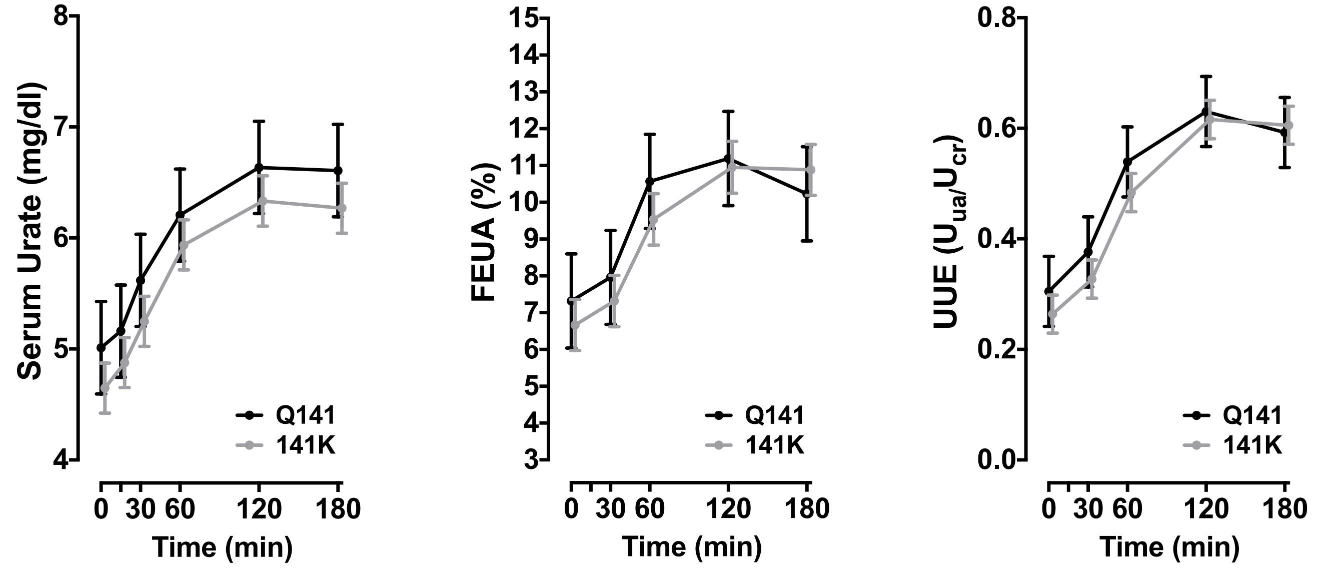

ANCOVA $P_{(time)}<0.0001$, $P_{(snp)}=0.0014$, $P_{(snp*time)}=0.99$ ANCOVA $P_{(time)}<0.0001$, $P_{(snp)}=0.26$, $P_{(snp*time)}=0.56$ ANCOVA $P_{(time)}<0.0001$, $P_{(snp)}=0.08$, $P_{(snp*time)}=0.66$

B: Analysis of non risk allele carriers (Q141) stratified by sex

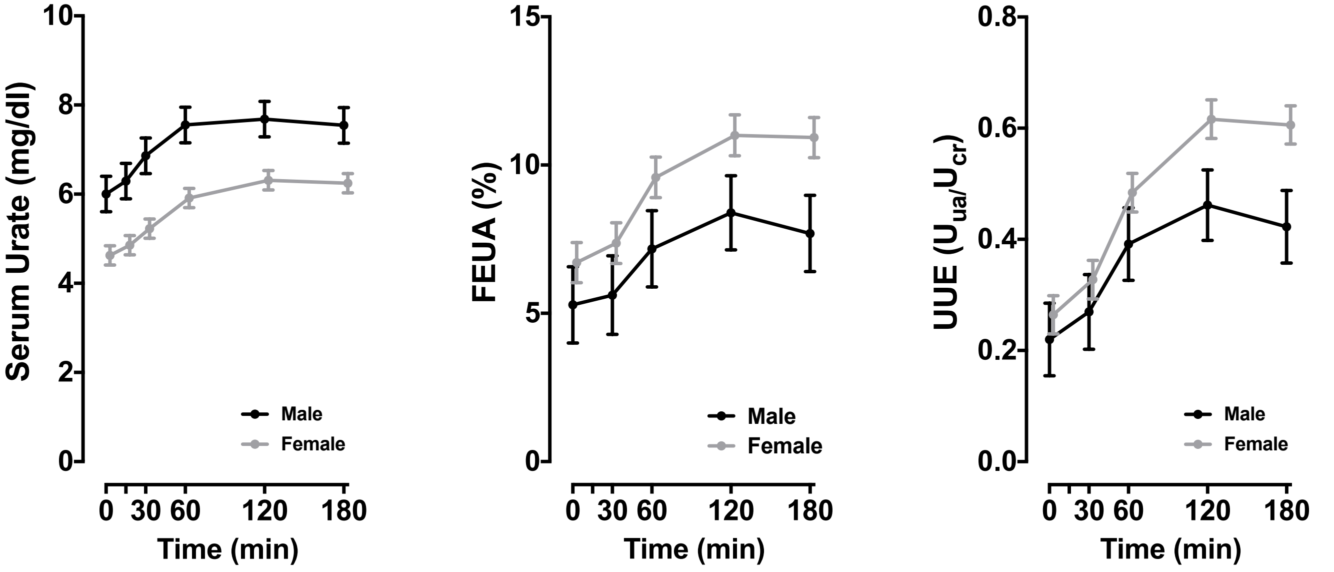

ANCOVA $P_{(time)}<0.0001$, $P_{(sex)}<0.0001$, $P_{(sex*time)}=0.86$ ANCOVA $P_{(time)}<0.0001$, $P_{(sex)}<0.0001$, $P_{(snp*time)}=0.45$ ANCOVA $P_{(time)}<0.0001$, $P_{(sex)}<0.0001$, $P_{(sex*time)}=0.038$

Supplementary Figure 7: Partial sex stratified analysis

A) Effect of *ABCG2* genotypes (Supplementary Table 1 for characteristics of participants), stratified by sex, on dependence of serum urate (SU), FEUA, and urinary urate excretion (UUE) in female only participants following inosine load (79 participants; +/- SD of mean). Statistical significance evaluated by a two-tailed ANCOVA, adjusted for age, ancestry, and BMI. (B) Effect of sex (non- risk allele only), on serum urate (SU), FEUA, and urinary urate excretion (UUE) in participants following inosine load (79 participants; +/- SD of mean). Statistical significance evaluated by a two-tailed ANCOVA, adjusted for age, ancestry, and BMI. Source data are provided as a Source Data file.

Supplementary Table 1. Characteristics of participants at baseline (n = 100). Unless stated, data are presented as mean (SD).

Age, years	29 (13)
Male sex, n (%)	21 (21%)
Ancestry	
Polynesian	50 (50%)
European	50 (50%)
Body mass index, kg/m^2	26 (6.0)
Waist circumference, cm	85 (13)
Systolic blood pressure, mmHg	117 (13)
Diastolic blood pressure, mmHg	67 (8)
Serum urate (mg/dL)	4.99 (1.31)
Serum creatinine (mg/dL)	0.79 (0.12)
Fractional excretion of urate, %	6.5 (2.4)
SLC2A9 rs11942223 protective C allele present, n (%)	38 (38%)
ABCG2 rs2231142 risk T allele present, n (%)	21 (21%; MAF 0.11)
ABCG2 rs2231142 risk T allele frequency by Ancestry	
Polynesian	13 (MAF: 0.13)
European	8 (MAF: 0.09)

Supplementary Table 2. Blood and urine chemistries of Female WT and Q140K+/+ mice

	Blood Chemistry WT (n=6)	Q141K+/+ (n=6)
**Na+ [mmol/l]**	132.50 ± 5.0	140.33 ± 2.63
**K+ [mmol/l]**	4.48 ± 0.30	4.60 ± 0.33
**Cl- [mmol/l]**	114.0 ± 1.24	113.0 ± 1.24
**BUN [mg/dl]**	21.33 ± 1.80	21.67 ± 0.92
**Hct [%PCV]**	31.0 ± 1.53	33.33 ± 1.15
**pH**	7.15 ± 0.03	**7.22 ± 0.02 [p=0.04]**
**PCO2 [mmHg]**	51.77 ± 5.43	50.10 ± 2.93
**HCO3 [mmol/l]**	17.62 ± 0.82	**20.47 ± 0.68 [p=0.011]**
**Beecf [mmol/l]**	-11.17 ± 0.60	**-7.17 ± 0.79 [p=0.001]**
**AnGap [mmol/l]**	7.17 ± 3.52	12.00 ± 2.10
**Hb [g/dl]**	10.57 ± 0.52	11.33 ± 0.38
	**Serum Chemistry**	
**IGF1 [pg/ml]**	150.03 ± 29.59	147.92 ± 36.54
**Insulin [ng/ml]**	1.378 ± 0.21	1.24 ± 0.29
**Glucose [mg/dl]**	175.67 ± 20.41	177.50 ± 14.41

	Urine Chemistry WT (n=6)	Q141K+/+ (n=6)
**Na+ [mmol/l]**	39.68 ± 2.33	34.85 ± 4.09
**K+ [mmol/l]**	115.83 ± 6.54	132.48 ± 6.76
**Cl- [mmol/l]**	114.4 ± 5.27	107.68 ± 9.8
**UV [ml/24hrs]**	3.62 ± 0.13	2.69 ± 0.46

Blood, serum, and urine chemistries from WT and Q140K+/+ female animals. Blood chemistries aquired using the iSTAT device (see methods), abbreviations as follows: BUN, Blood urea nitrogen; Hct, hematocrit; Beecf, Base excess; AnGap, anion gap; Hb, hemoglobin; IGF1, insulin like growth factor 1; UV, urinary volume (rate); Bold values are significant; Statistical analysis: one-tailed Student's t-tests, significance, +/-SEM.

Supplementary Table 3: Sex stratified analysis

ABCG2 rs2231142 risk T allele			
	**absent**	**present**	**Total**
Female	61	18	79
Male	18	3	21
Total	79	21	100

Model 1 Whole Cohort			
	**Serum Urate**	**FEUA (%)**	**UUE (Uua/Ucr)**
time	<0.0001	<0.0001	<0.0001
SNP	0.0005	0.82	0.12
*time*SNP*	0.99	0.69	0.86
Ethnicity	0.0014	0.0002	<0.0001
Sex	<0.0001	<0.0001	<0.0001
age	<0.0001	<0.0001	0.28
BMI	<0.0001	<0.0001	0.033

Model 2 Whole Cohort with Sex by SNP interaction Term			
	**Serum Urate**	**FEUA (%)**	**UUE (Uua/Ucr)**
time	<.0001	<.0001	<.0001
SNP	0.0226	0.2239	0.6574
*time*SNP*	0.9984	0.9874	0.9964
sex	<.0001	<.0001	<.0001
*Time*sex*	0.9286	0.9279	0.5713
*SNP*Sex*	0.7086	0.0447	0.3091
*time*SNP*Sex*	0.9974	0.8934	0.8761
Ethnicity	0.0016	0.0002	<.0001
age	<.0001	<.0001	0.2812
BMI	<.0001	<.0001	0.0288

Model 3 Just Females			
	**Serum Urate**	**FEUA (%)**	**UUE (Uua/Ucr)**
time	<.0001	<.0001	<.0001
SNP	0.0014	0.2552	0.0773
*time*SNP*	0.9995	0.558	0.6634
Ethnicity	<.0001	<.0001	0.0001
age	<.0001	<.0001	0.069
BMI	<.0001	<.0001	0.124
