# Supplementary material for: The ABCG2 Q141K hyperuricemia and gout associated variant illuminates the physiology of human urate excretion
Source: Nat Commun. 2020 Jun 2;11:2767. doi: 10.1038/s41467-020-16525-w (PMC7265540; doi:10.1038/s41467-020-16525-w)
Supplement: Supplementary file 3 — Source Data [file 41467_2020_16525_MOESM3_ESM.zip › 211133_3_rel_ms_0_q9x9yf.pdf]

PROTOCOL: THE INFLUENCE OF GENETIC VARIANTS ON INOSINE-INDUCED HYPERURICAEMIA

Investigators: **Prof Nicola Dalbeth MD FRACP**

Department of Medicine

University of Auckland

Prof Lisa Stamp PhD FRACP

Department of Medicine

University of Otago

Gregory Gamble MSc

Department of Medicine

University of Auckland

Prof Tony Merriman PhD

Department of Biochemistry

University of Otago

CONFIDENTIAL: Property of the Auckland Bone and Joint Research Group

1. AIMS

1.1 To determine the influence of *SLC2A9, ABCG2* and other genetic variants on inosine-induced hyperuricaemia.

1.2 To determine the influence of *SLC2A9, ABCG2* and other genetic variants on renal urate transport following inosine ingestion.

2. BACKGROUND

Gout is a disorder of purine metabolism, caused by precipitation of monosodium urate (MSU) crystals. This disease is of particular relevance to New Zealand, due to the high prevalence of severe, early onset gout in Māori and Pacific people. The central biochemical cause of gout is hyperuricaemia (elevated serum urate concentration). A number of checkpoints exist in regulation of serum urate concentrations. Key checkpoints are hepatic urate production from purine precursors, gut excretion an renal excretion.

Inosine is a purine nucleoside that is metabolised *in vivo* from AMP as part of the purine salvage metabolic pathway (Figure). It is degraded to hypoxanthine, which is, in turn, metabolised to xanthine and then urate in the purine degradation pathway. Oral supplementation of inosine is currently under clinical investigation for neuroprotection in multiple sclerosis and Parkinson's disease [1-3], due to its ability to increase serum urate concentrations. Administration of inosine leads to variable increases in serum urate concentrations [1]. **Thus oral administration of a fixed dose of inosine allows analysis of the effects of a purine load on both serum urate concentrations and renal uric acid handling.**

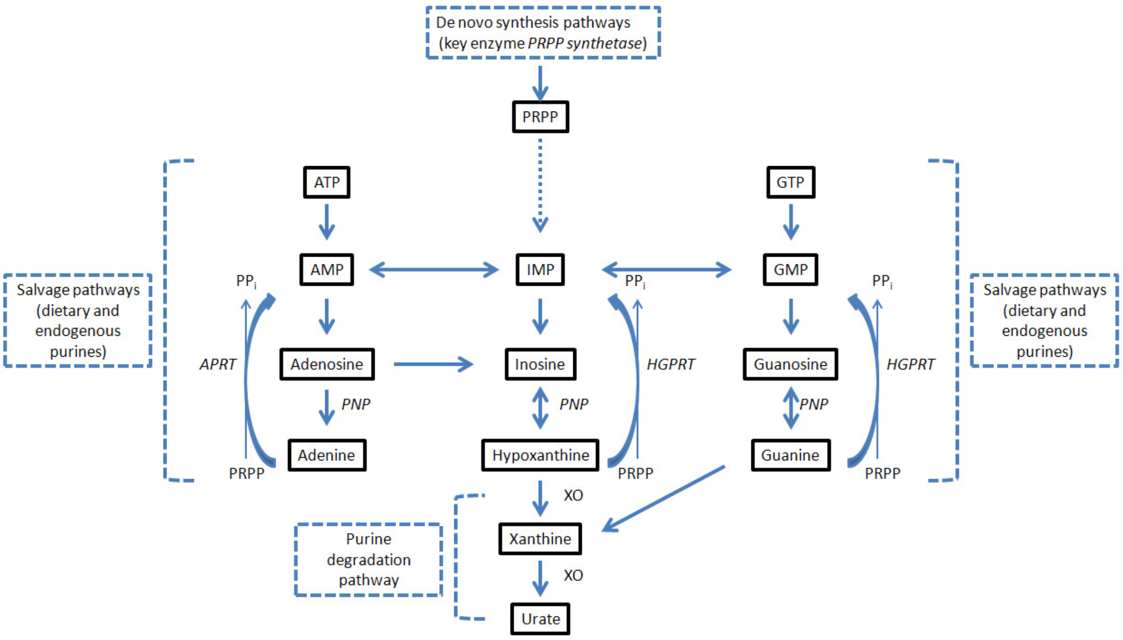

Figure 1. Purine synthesis and degradation pathways

The genome-wide association studies (GWAS) of serum urate and fractional excretion of uric acid (FEUA) have consistently reported a strong association with common variants of *SLC2A9* and *ABCG2* [4-8]. The Genetics of Gout in Aotearoa study has strongly implicated the *SLC2A9* gene in development of gout in Māori and Pacific people (odds ratios ≥5.0) [9]. Variation in the *rs11942223 SLC2A9* single nucleotide polymorphisms (SNP) was most strongly associated with gout in Māori, Pacific and Caucasian case-control cohorts in this study. Between 31.7%-47.2% of control participants possessed at least one protective alleles (i.e. 1/2 and 2/2 genotypes)

ABCG2 encodes the cellular efflux pump breast cancer resistance protein (BCRP). An *ABCG2* variant (*rs2231142, Q141K*) is strongly associated with gout and hyperuricaemia, and is thought to regulate serum urate concentrations through extra-renal (gut) underexcretion [10]. In addition, *ABCG2* variants play a role in treatment responses to allopurinol (a purine xanthine oxidase inhibitor), and BCRP can transport allopurinol [11].

Our recent studies of acute fructose loading have shown that *SLC2A9* and *ABCG2* variants influence changes in serum urate, serum glucose changes and fractional excretion of uric acid (FEUA) in response to a fructose load [12, 13]. It is unknown whether these effects occur due to direct interactions with fructose, or whether these effects are non-specific responses to hyperuricaemia induced by fructose. Here, we aim to examine the role of *SLC2A9* and *ABCG2* variants on serum urate responses and renal urate handling in response to an acute inosine load.

PRIMARY HYPOTHESIS

That individuals possessing at least one protective allele at SLC2A9 *rs11942223* have a lower hyperuricaemic response to an inosine challenge.

KEY SECONDARY HYPOTHESIS

That individuals possessing at least one *ABCG2 141K* variant have a higher hyperuricaemic response to an inosine challenge.

3. STUDY SYNOPSIS

Intervention study to examine the influence of genetic variation on inosine-induced hyperuricaemia. The primary endpoint is change in serum urate over 180 minutes after inosine ingestion. Key secondary and exploratory endpoints are change in fractional excretion of urate. The key genes of interest are *SLC2A9* (GLUT9) and *ABCG2.* However, other genes associated with purine synthesis and urate transport may also be examined in later exploratory analyses. This protocol is modified from previous our previous short term studies investigating the acute effect of skim milk and fructose on urate concentrations and renal excretion of uric acid in healthy volunteers [13, 14].

4. PARTICIPANT SELECTION

4.1. Summary

100 healthy participants without documented gout (50 of European Caucasian ancestry and 50 of Māori or Pacific ancestry.

4.2. Recruitment

Participants will be recruited by public advertising.

4.3. Inclusion criteria

a. Able to provide written informed consent

b. eGFR>60 mL/min/1.73 m^2

4.4. Exclusion criteria

a. History of gout

b. History of kidney stones

c. History of diabetes mellitus

d. Diuretic use

e. Urine pH ≤5.0 (risk factor for uric acid urolithiasis)

5. STUDY ENDPOINTS

5.1. Primary Endpoint

Change from baseline in serum urate concentration over 3 hours following an inosine challenge

5.2. Secondary Endpoint

Change in fractional excretion of urate to 3 hours following an inosine challenge. (The fractional excretion of urate is the ratio between the renal clearance of urate and the renal clearance of creatinine, expressed as a percentage).

6. STUDY VISITS

6.1. Summary of study visits

The protocol describes two visits. The study visit will occur within two weeks of the screening visit. The screening visit and study visit may be completed on the same day, depending on participant preference.

6.2. Visit 1: Screening visit

a. Discuss study and obtain written informed consent

b. Check inclusion and exclusion criteria met.

6.3. Visit 2: study visit summary

At the study visit, a venous catheter will be inserted for blood collection. Following an overnight fast, subjects will consume the inosine tablets (1.5g, Source Naturals Inosine) with 500ml water between 0800 and 0900. Blood will be obtained for urate, creatinine, and serum storage, prior to ingestion and then 15 minutes, 30 minutes, 60 minutes, 120 minutes, and 180 minutes after ingestion. Blood will also be obtained for DNA extraction at baseline. Urine volume will be measured and urine will be obtained at these time points (with the exception of 15 minutes post-ingestion) for testing of urate and creatinine. Participants will remain otherwise fasted for the remainder of the study visit and will be given a light meal after removal of the intravenous catheter.

6.4. Visit 2: study visit timeline

Time	Baseline (0800-0900)	Product ingestion	15 minutes from ingestion	30 minutes from ingestion	60 minutes from ingestion	120 minutes from ingestion	180 minutes from ingestion
**Insertion of iv cannula**	x						
**Completed intake of inosine**		x					
**Blood sample**	30ml		15ml	15ml	15ml	15ml	15ml
**Urine sample**	x			x	x	x	x
**Water ingestion**		500ml		Equivalent to urine volume	Equivalent to urine volume	Equivalent to urine volume	Equivalent to urine volume

6.5. Visit 2: Study visit details

a. Baseline data obtained

- History: past medical history, medications, allergies, alcohol history
- Family history of gout (detailed)
- Ancestry (self-reported, and recording of the ancestry of each grandparent)
- Clinical examination: weight, height, waist circumference and blood pressure

b. Confirm fasting status

c. Venous catheter inserted

d. Baseline blood and urine samples obtained including blood for DNA extraction

e. Inosine tablets taken with 500ml water

f. Blood samples obtained at 15, 30, 60, 120 and 180 minutes

CONFIDENTIAL: Property of the Auckland Bone and Joint Research Group

g. Urine samples obtained at 30, 60, 120 and 180 minutes

h. Water ingestion (volume equivalent to urine volume) at 30, 60, 120 and 180 minutes

i. Venous catheter removed

j. Light meal given

7. TEST SUBSTANCE

The test substance is inosine tablets (3x500mg tablets, Source Naturals Inosine). Each tablet supplement contains 500mg inosine, 30mg calcium, plus dibasic calcium phosphate, sorbitol, stearic acid, modified cellulose gum and magnesium stearate. This supplement is available from Health Food suppliers. The dose of 1.5g is consistent with doses used in other clinical trials of inosine [1, 2] and is within the recommended daily dose range. In a long-term study of inosine in Parkinson's disease, a mean (SD) urate increase of 0.18 (0.07) mmol/L was observed with a mean dose of 1.53g inosine [1].

8. LABORATORY TESTING

8.1. Baseline blood sample

a. Urate

b. Creatinine

c. Glucose

d. Plasma stored for purine and other metabolites

e. DNA for testing of *SLC2A9* and *ABCG2* variants, other genes involved in purine synthesis and urate transport

8.2. Blood samples at all other times points

a. Urate

b. Creatinine

c. Glucose

d. Plasma stored for purine and other metabolites

8.3. Urine samples at baseline and all other timepoints (except 15 minutes post ingestion)

a. Urate

b. Creatinine

c. Volume recorded

d. Urine stored for purine and other metabolites

9. STATISTICAL CONSIDERATIONS

9.1. Justification of Sample Size and Study Population

We plan to recruit 100 healthy subjects without documented gout; 50 participants of Māori or Pacific ancestry and 50 participants of Caucasian ancestry. We have chosen to pool Māori and Pacific participants into a single group of Polynesian ancestry, as the allele frequencies for *SLC2A9* are very similar in these two groups, and our previous data also indicate similar renal responses to hyperuricaemia in Māori and Pacific participants [13]. Sample size calculations have been based on the variability of change in serum urate at each time point in our previous study of fructose induced hyperuricaemia [13] and an analysis of inosine use for Parkinson's disease [1]. We have selected the *SLC2A9* SNP *rs11942223* for the primary analysis, and assume that approximately 30% of participants will have the protective allele based on our previous work in the Genetics of Gout in Aotearoa study [9]. The variability in the change in serum urate to fructose load [13] was consistent within groups with and without protective alleles across all time points (SD 0.03 mmol/l). With 70 participants without the protective allele and 30 with the protective allele the proposed study is adequately powered (90% at the 5% significance level for a two tailed test) to detect a difference in the change in serum urate between these groups of at least 0.02mmol/l.

We wish to perform a subgroup analysis using Māori or Pacific and Caucasian case-control cohorts, and have powered the study based on the above assumptions. Here we will recruit 50 participants of each ancestry, and estimate that 14-16 participants within each ancestral group will have the protective allele. In the smallest group comparison, n=16 vs. n=34 within an ancestral subgroup, there is in excess of 90% power (at the 5% significance level for a two tailed test) to detect a difference in the change in serum urate 180 minutes after an oral inosine challenge of at least 0.03 mmol/l. Pooled factorial secondary analyses will therefore similarly be adequately powered for the same effect size.

Whilst the primary analysis will be performed on the change from baseline, no adequate estimate of the variability of this is available and so the conservative approach of modelling the difference between groups at a single time point was chosen to validate the sample size. Removing between patient variability by using the change from baseline as the dependent variable is likely to offer increased power which should more than offset the very small number of samples which may ot be adequately genotyped. Sample size calculations were performed using PASS 2002 (Hintze, J (2006) Kaysville, Utah).

9.2. Statistical Methods

Data will be presented as mean (SD) or median (IQR) for descriptive purposes. Measures of effect will be presented with the appropriate 95% confidence interval. The primary analysis will be a comparison of the hyperuricaemic response to an inosine challenge in the entire group (n=100), based on the presence or absence of the *rs11942223* protective allele. Secondary and exploratory analyses will be comparison of the hyperuricaemic response to an inosine challenge based on subgroup analysis (within each ancestral subgroup), and based on other clinical features, such as age, sex and BMI in the entire group.

A key secondary analysis is the effect of the *ABCG2 Q141K* risk variant on the serum urate and FEUA response to an inosine challenge.

Data will be analysed using a mixed models approach to repeated measures where the dependent variable, change from baseline, will be examined in an analysis of covariance. Main effects of allele presence/absence, time and their interaction will be constructed and the baseline value of the dependent variable will be included as covariate Significant group effects will be explored using the method of Tukey to preserve an overall 5% significance level. All analyses will be performed using SAS (SAS Institute Inc v 9.4) on an intention to treat basis. All tests will be two-tailed.

10. APPROVALS

Ethical approval for the study will be obtained from the Multi-Regional Ethics Committee (MREC) prior to commencement of the study. All patients will provide written informed consent prior to participation in the study.

11. STUDY PROTOCOL SUMMARY

Visit No.	1	2
Visit type	Screen	Study
Informed consent	√	
Inclusion/exclusion criteria	√	
Laboratory tests (eGFR, urine pH testing)	√	
Confirm fasting status		√
Relevant medical history recorded		√
Detailed family history recorded		√
Ancestry recorded		√
Weight, height, waist circumference		√
Blood pressure		√
Insert venous catheter		√
Baseline blood and urine testing, including for DNA		√
Administer inosine tablets		√
Serial blood and urine testing		√
Remove venous catheter		√
Record adverse events		√

12. REFERENCES

1 Parkinson Study Group Sure-PD Investigators, Schwarzschild MA, Ascherio A, et al. Inosine to increase serum and cerebrospinal fluid urate in Parkinson disease: a randomized clinical trial. JAMA Neurol 2014;71:141-50.
2 Markowitz CE, Spitsin S, Zimmerman V, et al. The treatment of multiple sclerosis with inosine. J Altern Complement Med 2009;15:619-25.
3 Gonsette RE, Sindic C, D'Hooghe M B, et al. Boosting endogenous neuroprotection in multiple sclerosis: the ASsociation of Inosine and Interferon beta in relapsing- remitting Multiple Sclerosis (ASIIMS) trial. Mult Scler 2010;16:455-62.
4 Li S, Sanna S, Maschio A, et al. The GLUT9 gene is associated with serum uric acid levels in Sardinia and Chianti cohorts. PLoS Genet 2007;3:e194.
5 Dehghan A, Kottgen A, Yang Q, et al. Association of three genetic loci with uric acid concentration and risk of gout: a genome-wide association study. Lancet 2008;372:1953-61.
6 Kolz M, Johnson T, Sanna S, et al. Meta-analysis of 28,141 individuals identifies common variants within five new loci that influence uric acid concentrations. PLoS Genet 2009;5:e1000504.
7 Doring A, Gieger C, Mehta D, et al. SLC2A9 influences uric acid concentrations with pronounced sex-specific effects. Nat Genet 2008;40:430-6.
8 Kottgen A, Albrecht E, Teumer A, et al. Genome-wide association analyses identify 18 new loci associated with serum urate concentrations. Nat Genet 2013;45:145-54.
9 Hollis-Moffatt JE, Xu X, Dalbeth N, et al. Role of the urate transporter SLC2A9 gene in susceptibility to gout in New Zealand Maori, Pacific Island, and Caucasian case-control sample sets. Arthritis Rheum 2009;60:3485-92.
10 Ichida K, Matsuo H, Takada T, et al. Decreased extra-renal urate excretion is a common cause of hyperuricemia. Nat Commun 2012;3:764.
11 Wen CC, Yee SW, Liang X, et al. Genome-wide association study identifies ABCG2 (BCRP) as an allopurinol transporter and a determinant of drug response. Clin Pharmacol Ther 2015;97:518-25.
12 Dalbeth N, House ME, Gamble GD, et al. Influence of the ABCG2 gout risk 141 K allele on urate metabolism during a fructose challenge. Arthritis Res Ther 2014;16:R34.
13 Dalbeth N, House ME, Gamble GD, et al. Population-specific influence of SLC2A9 genotype on the acute hyperuricaemic response to a fructose load. Ann Rheum Dis 2013;72:1868-73.
14 Dalbeth N, Wong S, Gamble GD, et al. Acute effect of milk on serum urate concentrations: a randomised controlled crossover trial. Ann Rheum Dis 2010;69:1677-82.

CONFIDENTIAL: Property of the Auckland Bone and Joint Research Group
